# Supplementary figures and images for: Effects of regional limb perfusion technique on concentrations of antibiotic achieved at the target site: A meta-analysis
Source: PLoS One. 2022 Apr 1;17(4):e0265971. doi: 10.1371/journal.pone.0265971 (PMC8974993; doi:10.1371/journal.pone.0265971)

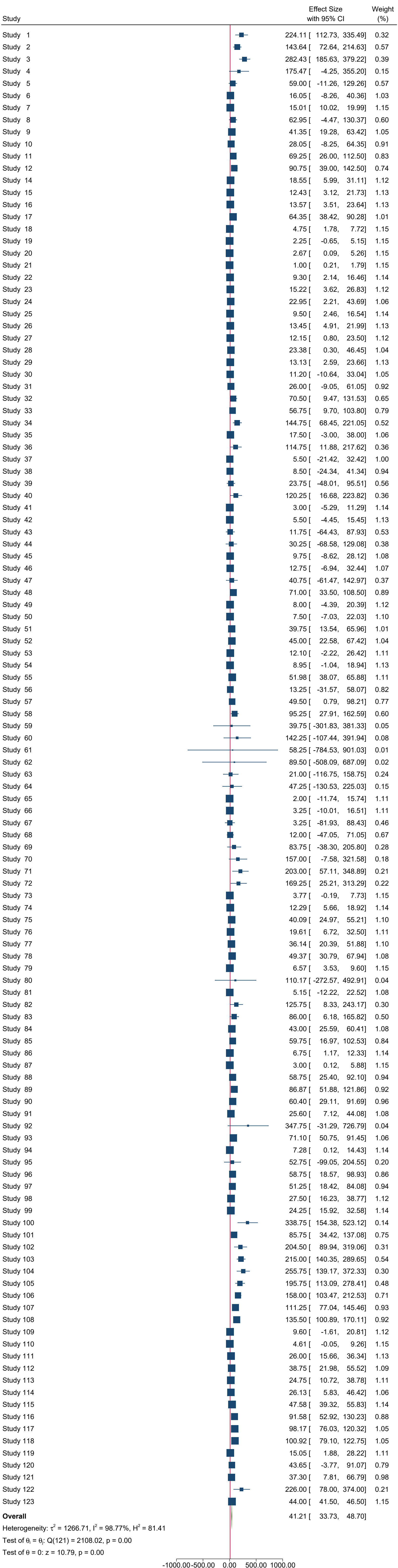

Supplement: S1 Fig — A vertical red line depicts 10x MIC. (PDF) [file pone.0265971.s001.pdf]

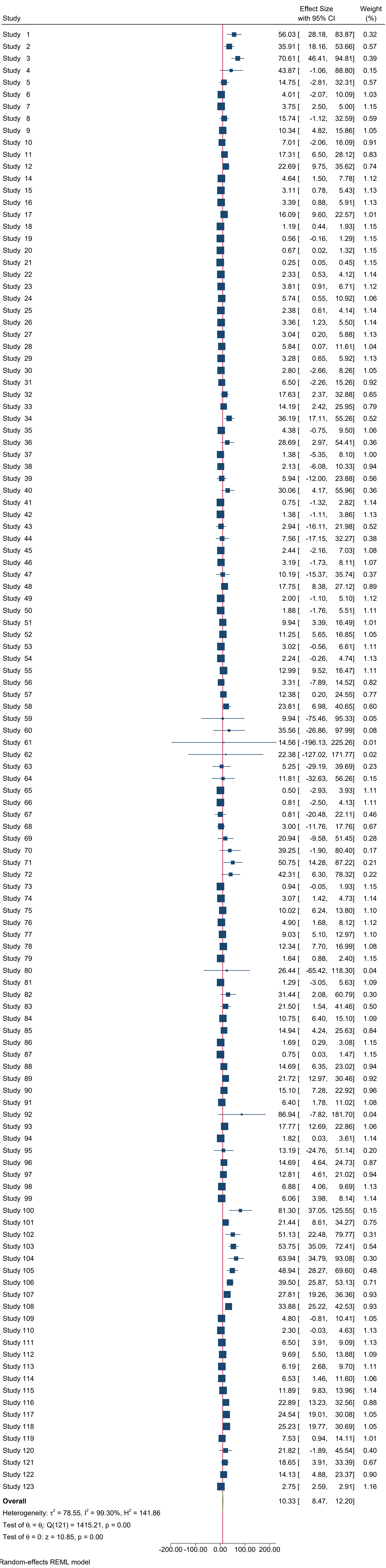

Supplement: S2 Fig — A vertical red line depicts 10x MIC. (PDF) [file pone.0265971.s002.pdf]
