# Supplementary material for: Effects of regional limb perfusion technique on concentrations of antibiotic achieved at the target site: A meta-analysis
Source: PLoS One. 2022 Apr 1;17(4):e0265971. doi: 10.1371/journal.pone.0265971 (PMC8974993; doi:10.1371/journal.pone.0265971)
Supplement: S2 Table — Modified from Wan et al. 2014. [14]. (DOCX) [file pone.0265971.s004.docx]

**S2 Table. Values of *ξ (n)* in the formula *S ≈ b - a* and the formula *S ≈* ½ (*b – a* + *q_3_ – q_1_*)**

***ξ (n) ξ (n) η (n)***

**for *n ≤* 20.** Modified from Wan et al. 2014.

| *n* | *ξ (n)* | *n* | *ξ (n)* |
| --- | --- | --- | --- |
| 1 | 0 | 11 | 3.173 |
| 2 | 1.128 | 12 | 3.259 |
| 3 | 1.693 | 13 | 3.336 |
| 4 | 2.059 | 14 | 3.407 |
| 5 | 2.326 | 15 | 3.472 |
| 6 | 2.534 | 16 | 3.532 |
| 7 | 2.704 | 17 | 3.588 |
| 8 | 2.847 | 18 | 3.640 |
| 9 | 2.970 | 19 | 3.689 |
| 10 | 3.078 | 20 | 3.735 |
