# Supplementary material for: Effects of regional limb perfusion technique on concentrations of antibiotic achieved at the target site: A meta-analysis
Source: PLoS One. 2022 Apr 1;17(4):e0265971. doi: 10.1371/journal.pone.0265971 (PMC8974993; doi:10.1371/journal.pone.0265971)
Supplement: S3 Table — Modified from Wan et al. 2014. [14]. (DOCX) [file pone.0265971.s005.docx]

**S3 Table. Values of *η (n)* in the formula *S ≈* ½ (*b – a* + *q_3_ – q_1_*) and the formula**

***ξ (n) η (n)***

***S ≈ q_3_ – q_1_* for Q ≤ 20, where *n* = 4*Q* + 1.** Modified from Wan et al. 2014.

***η (n)***

| *Q* | *η (n)* | *Q* | *η (n)* |
| --- | --- | --- | --- |
| 1 | 0.990 | 11 | 1.307 |
| 2 | 1.144 | 12 | 1.311 |
| 3 | 1.206 | 13 | 1.313 |
| 4 | 1.239 | 14 | 1.316 |
| 5 | 1.260 | 15 | 1.318 |
| 6 | 1.274 | 16 | 1.320 |
| 7 | 1.284 | 17 | 1.322 |
| 8 | 1.292 | 18 | 1.323 |
| 9 | 1.298 | 19 | 1.324 |
| 10 | 1.303 | 20 | 1.326 |
